# Subungual Myiasis Presenting to the Emergency Department: A Case Report

**DOI:** 10.5811/cpcem.53959

**Published:** 2026-06-29

**Authors:** Taylor D. Marquis, Alexander Y. Sheng

**Affiliations:** Brown University, Warren Alpert School of Medicine, Department of Emergency Medicine, Providence, Rhode Island

**Keywords:** emergency department, myiasis, subungual, case report

## Abstract

**Introduction:**

Subungual myiasis is an infection under the fingernail or toenail caused by an infestation of fly larvae. It is rarely reported internationally, with only one previously reported case in the United States.

**Case Report:**

An 80-year-old female with a history of polyneuropathy, peripheral artery disease, peripheral venous insufficiency, and chronic bilateral lower extremity edema presented to the emergency department (ED) after staff at her skilled nursing facility noted what appeared to be several whitish-colored maggots moving under the nail of the patient’s left great toe. Thorough examination was consistent with subungual myiasis with associated onycholysis without evidence of associated cellulitis or soft tissue infection. Manual extraction of six larvae was performed, and the patient’s foot was soaked in chlorhexidine. She was discharged back to her facility with a plan for daily chlorhexidine soaks and prompt follow-up with a podiatrist. To date she has not presented to the ED since with the same issue.

**Conclusion:**

Due to its extraordinarily rare incidence, there is little consensus regarding the treatment of subungual myiasis beyond the manual extraction of larvae. Oral ivermectin has been reported as an additional treatment option for myiasis, but research is limited. Antibiotics are generally only recommended when there is evidence of associated bacterial infection. This case should raise awareness for emergency physicians regarding the recognition and management of subungual myiasis.

## INTRODUCTION

Myiasis is a parasitic infection caused by the infestation of living tissue by fly larvae. Cutaneous and wound myiasis are the most reported manifestations of the disease, although many other forms exist.^1^ Subungual myiasis, in which myiasis develops under a fingernail or toenail, is a rarely reported disease worldwide. A thorough literature search yielded only a single case report of subungual myiasis in the United States.^2^ Since 1980, seven cases have been reported internationally in Spain, Turkey, Italy, South Korea, and Syria.^3^–^8^ All patients had predisposing risk factors including peripheral venous insufficiency and lower extremity edema,^2^ poor personal hygiene,^4^,^7^ immunosuppressive drugs including chemotherapy,^5^–^7^ or an ingrown toenail. There was substantial treatment variation between these seven patients. We present the second reported case of a patient with subungual myiasis in the United States.

## CASE REPORT

An 80-year-old female with a history of hypertension, hyperlipidemia, polyneuropathy, peripheral artery disease, peripheral venous insufficiency status post bilateral iliac vein stenting and chronic bilateral lower extremity edema presented to an urban emergency department (ED) in New England during the summer with a chief complaint of a nail problem. The patient was residing at a skilled nursing facility due to difficulties performing independent activities of daily living. She had no history of recent international travel and was not taking immunosuppressive medications or undergoing chemotherapy. She was sent to the ED by facility staff who noted what appeared to be several whitish-colored maggots, or larvae, moving under the nail of the patient’s left great toe while performing routine care. The patient denied pain, abnormal sensation, or any awareness of the issue.

On physical examination, the distal end of a discolored nail plate was raised from the nail bed. Multiple writhing larvae were visible between the hyponychium and the nail plate. These findings were consistent with subungual myiasis with associated onycholysis. There was no evidence of associated cellulitis or soft tissue infection (Video 1).

Following exam, the patient’s left foot was soaked in a chlorhexidine solution. Six, approximately 1-cm long, live larvae were extracted from under the patient’s left great toenail using forceps (Video 2).

The larvae were not sent for speciation but did have characteristics classic of Sarcophagidae (Video 3).^9^

The patient’s left foot was again soaked in chlorhexidine, and no other maggots or larvae were noted on a thorough re-examination. She was discharged back to her facility with a plan for daily chlorhexidine soaks and prompt follow-up with a podiatrist, with whom she was already established as a patient. Per chart review, the patient did not represent to an ED in the same medical system within 60 days of discharge for the same issue.

## DISCUSSION

Sarcophagidae, commonly known as flesh flies, produce larvae that are necrophagous. They remain in larval stage for 16–30 days, during which time they spend 5–10 days feeding on decomposing mammalian tissue.^10^ Myiasis of all types is most diagnosed in warm, tropical or subtropical climates, and is relatively rare in the United States.^1^ Our patient’s main risk factors for the development of subungual myiasis included peripheral artery disease, peripheral venous insufficiency requiring iliac vein stenting, and chronic lower extremity edema. She had no history of recent international travel, and she was not taking immunosuppressive medications or undergoing chemotherapy.

Point-of-care ultrasound using high-frequency linear probe can be used to exclude abscess and visualize hyperechoic mobile structures indicative of larvae.^2^ Due to the rarity of such cases, there is no guidance on whether specimens should go for speciation, especially as doing so may not change management. All seven previously reported cases of subungual myiasis were treated with manual removal of the larvae.^3^–^8^ Nail extraction was performed in five of the previously reported cases.^3^,^4^,^6^–^8^ This was not deemed necessary in our patient’s case as her associated mild onycholysis allowed for full visualization of the area and extraction of the larvae without removal of the healthy-appearing nail plate. One case was additionally treated with footbaths containing an unspecified antiseptic solution in addition to both oral and topical antibiotics.^5^ Two other cases were also treated with oral antibiotics.^4^,^7^

*CPC-EM Capsule*

What do we already know about this clinical entity?

*Myiasis is a parasitic infection caused by fly larvae infestation of living tissue.*

What makes this presentation of disease reportable?

*This is only the second published case report of subungual myiasis in the United States.*

What is the major learning point?

*There is little consensus regarding the treatment of subungual myiasis beyond manual extraction of larvae.*

How might this improve emergency medicine practice?

*Given the rise in international travel and changing climate, emergency physicians should recognize the possibility of myiasis in all its manifestations.*

Due to its rare incidence, there is little consensus regarding the treatment of subungual myiasis beyond the manual extraction of larvae. Oral ivermectin has been reported as an additional treatment option for myiasis, but this is generally limited to veterinary studies or individual case reports in humans.^11^,^12^ Topical and systemic antibiotics are generally only recommended when there is evidence of associated bacterial infection.^1^ Given the rise of international travel and a changing climate, emergency physicians in the United States should be aware of such cases and be able to recognize and treat myiasis in all its manifestations, including subungual myiasis. Further research into treatments for myiasis are needed as there is little consensus on the role of systemic antiparasitic medications such as ivermectin.

## CONCLUSION

Subungual myiasis is a rare, although likely under-reported, disease in the United States. Risk factors include peripheral venous insufficiency and lower extremity edema, poor personal hygiene, immunosuppressive drugs and trauma to the area. It can be effectively treated in the ED or in the outpatient clinic setting. Manual removal of the larvae remains the mainstay of treatment, but the use of treatment adjuncts such as antiparasitic medications remains controversial. Due to variations in practice, further research on the best course for treatment for subungual myiasis is needed.

## Supplementary Information

Video 1 Larvae moving under patient’s left great toenail, consistent with subungual myiasis.

Video 2 Extraction of larvae from under patient’s left great toenail using forceps.

Video 3 Six, approximately 1-cm long larvae extracted from under patient’s toenail with characteristics classic of Sarcophagidae.
